# Characterization of Common Carp Transcriptome: Sequencing, *De Novo* Assembly, Annotation and Comparative Genomics

**DOI:** 10.1371/journal.pone.0035152

**Published:** 2012-04-13

**Authors:** Peifeng Ji, Guiming Liu, Jian Xu, Xumin Wang, Jiongtang Li, Zixia Zhao, Xiaofeng Zhang, Yan Zhang, Peng Xu, Xiaowen Sun

**Affiliations:** 1 The Centre for Applied Aquatic Genomics, Chinese Academy of Fishery Sciences, Beijing, China; 2 CAS Key Laboratory of Genome Sciences and Information, Beijing Institute of Genomics, Chinese Academy of Sciences, Beijing, China; 3 Heilongjiang Fisheries Research Institute, Chinese Academy of Fishery Sciences, Harbin, China; Auburn University, United States of America

## Abstract

**Background:**

Common carp (*Cyprinus carpio*) is one of the most important aquaculture species of Cyprinidae with an annual global production of 3.4 million tons, accounting for nearly 14% of the freshwater aquaculture production in the world. Due to the economical and ecological importance of common carp, genomic data are eagerly needed for genetic improvement purpose. However, there is still no sufficient transcriptome data available. The objective of the project is to sequence transcriptome deeply and provide well-assembled transcriptome sequences to common carp research community.

**Result:**

Transcriptome sequencing of common carp was performed using Roche 454 platform. A total of 1,418,591 clean ESTs were collected and assembled into 36,811 cDNA contigs, with average length of 888 bp and N50 length of 1,002 bp. Annotation was performed and a total of 19,165 unique proteins were identified from assembled contigs. Gene ontology and KEGG analysis were performed and classified all contigs into functional categories for understanding gene functions and regulation pathways. Open Reading Frames (ORFs) were detected from 29,869 (81.1%) contigs with an average ORF length of 763 bp. From these contigs, 9,625 full-length cDNAs were identified with sequence length from 201 bp to 9,956 bp. Comparative analysis revealed that 27,693(75.2%) contigs have significant similarity to zebrafish Refseq proteins, and 24,371(66.2%), 24,501(66.5%) and 25,025(70.0%) to teraodon, medaka and three-spined stickleback refseq proteins. A total of 2,064 microsatellites were initially identified from 1,730 contigs, and 1,639 unique sequences had sufficient flanking sequences on both sides for primer design.

**Conclusion:**

The transcriptome of common carp had been deep sequenced, *de novo* assembled and characterized, providing the valuable resource for better understanding of common carp genome. The transcriptome data will facilitate future functional studies on common carp genome, and gradually apply in breeding programs of common carp, as well as closely related other Cyprinids.

## Introduction

Common carp (*Cyprinus carpio L*) is a member of Cyprinidae and natural distributes in Eurasia continent. It had been cultured in many countries in East Asia and Europe for several thousand years. In the past two centuries, common carp was introduced into Africa and America, which make it one of the most important aquaculture species worldwide with an annual global production of 3.4 million metric tons [1]. Although common carp is one of the most important food fish with over hundred strains and varieties in the world, it is also selected and kept for decorative purposes. The most famous and well-recognized ornamental common carp is koi which originally bred in Japan in the 1,820 s and gradually developed many varieties distinguished by coloration, patterning, and scalation. Similarly, local Chinese in Zhejiang Province bred Oujiang color carps with multiple color patterns for both ornamental and food purpose [2]. In addition to its economical importance, common carp is also considered as a model species for studies on immunology [3], ecology [4], environmental toxicology [5], [6], developmental biology [7], evolution [8].

Due to the economical and ecological importance of common carp, genetic studies had been performed in the past decade, which focused on development of genetic markers [3], [9]–[11] for breeding and genetic evaluation, construction of genetic maps [12], [13] and physical map [14], collection of a large set of ESTs [15]–[17] and microRNA [18], construction of bacterial artificial chromosome (BAC) library [19] and collection BAC-end sequences (BES) [20], transcriptome study with cDNA microarrays [21], characterization of functional genes [22] and quantitative trait loci (QTL) analysis [23], [24].

EST sequencing has been considered as an efficient approach for genomic study and functional gene identification, especially for those species without a genome sequence. In the past decade, tens of thousands ESTs had been developed on several important aquaculture speices with traditional Sanger's methods according to dbEST summary (http://www.ncbi.nlm.nih.gov/dbEST/dbEST_summary.html), including two catfish species (500,000) [25], Atlantic salmon (498,212), rainbow trout (287,967), Altantic cod (229,094) and pacific oyster (206,388), as well as some aquatic parasite species like *Ichthyophthirius multifiliis* (33,516) [26]. These EST resources allow efficient gene discovery and transcriptome profiling in these species [27]–[31], as well as comparative genome analysis with well-sequenced model species for better understanding the genomes of these aquaculture species. There are over 34,000 ESTs available for common carp in the Genbank ESTdb, collected by common carp research community in the past several years. However, it remains insufficient for the comprehensive understanding of common carp transcriptome. Many low expression transcripts or tissue-specific transcripts would be missed from current EST data, which makes it difficult for further analysis on transcriptome. More EST sequences and well assembled transcriptome sequences are desired to fulfill inclusive research of common carp. High throughput next generation sequencing technologies provide us the platforms to do sequence common carp transcriptome deeply with much lower cost than traditional Sanger method, which had boosted genetic and genomic research of relative lagging species [32]–[34].

In the present study, we performed *de novo* transcriptome sequencing of common carp using Roche 454 GS FLX platform. Over 1,418,591 clean ESTs were collected and assembled into 36,811 cDNA contigs. Annotation and gene ontology analysis were then performed on these contigs, providing the valuable resource for future genetic and genomic research on common carp and closely related species.

## Results and Discussion

### Generation of expressed short reads

Using Roche 454 sequencing technology, a total of 2,116,226 raw sequencing reads with average length of 331 bp were generated. The raw reads produced in this study have been deposited in the NCBI SRA database (accession number: SRA050545). After removal of ambiguous nucleotides, low-quality sequences (quality scores<20), contaminated microbial sequences, ribosomal RNA sequences, common carp mitochondrial genome sequences, a total of 1,418,591 cleaned reads ranging from 100 bp to 638 bp were harvested, with an average length of 321 bp and a median length of 328 bp (Table 1 and Figure S1).

**Table 1:** Statistics of common carp transcriptome sequences.

Number of raw reads	2,116,226
Average length of raw reads	331 bp
Number of cleaned reads	1,418,591
Average length of cleaned reads	321 bp
Median length of cleaned reads	328 bp
Sequences for assembly	1,150,339

### Assembly of common carp transcriptome

After BLASTed against zebrafish (*Danio Rerio*) protein database, the cleaned reads were divided into two groups with 778,472 reads in group 1and 640,119 reads in group 2. The reads in two groups were assembled separately with Newbler 2.5 and MIRA. Newbler 2.5 had produced 38,278 contigs and 34,005 singletons for group 1, 75,644 contigs and 74,475 singletons for group 2. A total of 93,631 contigs were then assembled with CAP3. Similarly, 40,570 contigs and 173,837 singletons were generated for group 1, and 74,050 contigs and 155,822 singletons were generated for group 2 by using MIRA. Then 52,346 contigs were assembled with CAP3. Re-assembling step with CAP3 removed the redundancy and harvested 41,509 contigs with higher reliability (Table S1). Custom perl scripts were then used to connect the contigs that were mapped on same zebrafish reference gene by adding 20 “X”, and we finally collected 36,811 contigs. The lengths of contig range from 100 to 14,971 bp, with the average length of 888 bp, N50 length of 1,002 bp and median length of 689 bp (Table 2 and Figure S2).

**Table 2:** Statistics of transcriptome assembly.

Contig number	36,811
Maximum contig length	14,971 bp
Minimum contig length	100 bp
Average contig length	888 bp
N50 length	1,002 bp
Number of reads per contig	31.3

### Functional annotation

**Figure 1:**
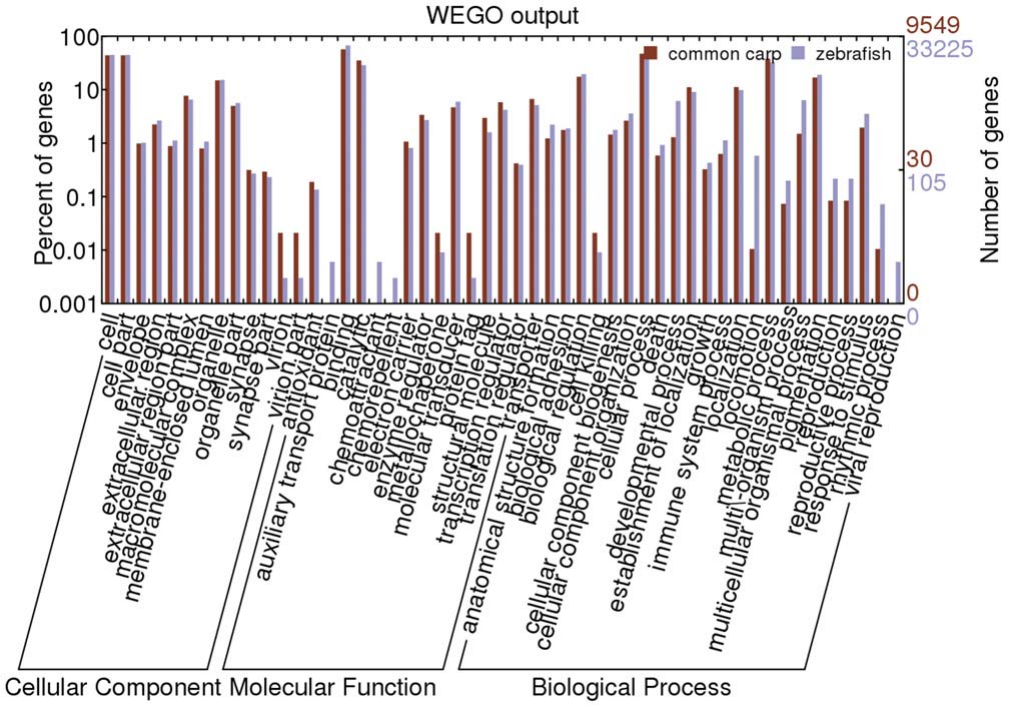
Comparative analysis and functional classification of common carp and zebrafish genes.

All assembled contigs were first compared with NCBI non-redundant (nr) protein database for functional annotation by using BLASTx with e-value cutoff of 1e-10. A total of 28,443 contigs had significant hit, corresponding to 19,165 unique protein accessions in the nr protein database (Table 3). The gene name of top best BLASTx hit was assign to each contigs with significant hits. Gene ontology (GO) analysis was conducted on those 19,165 unique proteins by using InterProScan (http://www.ebi.ac.uk/Tools/pfa/iprscan/) and integrated protein databases with default parameters. A total of 9,549 unique proteins were assigned at least one GO term for describing biological processes, molecular functions and cellular components. InterProScan output file was input into BGI WEGO program and GO annotations were plotted (http://wego.genomics.org.cn) (Figure 1). Of these, the molecular function ontology made up the majority (8258, 86.5%), followed by biological process (6025, 63.1%) and cellular component (4383, 45.9%). Briefly, for biological processes, genes involved in cellular process (GO: 0009987) and metabolic process (GO: 0008152) were highly represented; for molecular functions, binding (GO: 0005488) were the most represented GO term, followed by catalytic activity (GO: 0003824); cells (GO: 0005623) and organelles (GO: 0043226) were the most represented categories for cellular component. To assess the functional diversity of assembled transcriptome, GO annotations of zebrafish (Ensembl) were compared with those of common carp transcriptome (Figure 1), reflecting a similar functional distribution on GO categories and indicating the sequence diversity of the transcriptome study. The potions of virion (GO:0019012) and virion part (GO:0044423) in common carp were almost three times higher than those of zebrafish, suggesting that open-water environment may introduce more virus into tissues of common carp even we had performed data clean-up very carefully with available microbe sequences.

**Table 3:** Summary of BLASTX search results of common carp transcriptome.

Database	common carp hits	Unique protein	% of total unique proteins
NR	28,055	19,165	
Refseq/Ensembl			
Zebrafish	27,693	14,554	53.4% of 27,271
Medaka	24,501	12,471	50.6% of 24,661
Tetraodon	24,371	12,536	54.2% of 23,118
Three-spined stickleback	25,025	13,147	47.7% of 27,576

In addition, KEGG pathway analysis was performed on all assembled contigs as alternative approach for functional categorization and annotation. Enzyme commission (EC) numbers were assigned to 3,699 unique sequences, which categorized them into different functional groups (Table 4). Briefly, of these sequences with KEGG annotation, 1,143 (30.9%) were classified into the metabolism, including majority sub-groups of carbohydrate metabolism (906 sequences), amino acid metabolism (210 sequences) and lipid metabolism (248 sequences). Sequences grouped into the Genetic information processing (GIP), accounted for 1,098 (29.7%), including translation (426 sequences), folding, sorting and degradation (408 sequences), transcription (187 sequences) , replication and repair (129 sequences), etc. organismal systems ,cellular processes and environmental information processing (EIP) groups contained 1085(29.3%), 908(24.5%) and 775 (21.0%) KEGG annotated sequences, respectively.

**Table 4:** KEGG biochemical mappings for common carp.

KEGG categories represented	Unique sequences* (Number of KO)
**Metabolism**	**1,143 (849)**
Carbohydrate Metabolism	906 (682)
Amino Acid Metabolism	210 (169)
Energy Metabolism	174 (134)
Nucleotide Metabolism	144 (109)
Metabolism of Cofactors and Vitamins	117 (93)
Lipid Metabolism	248 (171)
Glycan Biosynthesis and Metabolism	149 (119)
Metabolism of Other Amino Acids	82 (55)
Xenobiotics Biodegradation and Metabolism	69 (51)
Biosynthesis of Secondary Metabolites	20 (17)
Biosynthesis of Polyketides and Nonribosomal Peptides	22 (21)
**Genetic Information Processing**	**1,098 (805)**
Replication and Repair	129 (101)
Folding, Sorting and Degradation	408 (307)
Transcription	187 (147)
Translation	426 (290)
**Environmental Information Processing**	**775 (532)**
Signal Transduction	564 (383)
Signaling Molecules and Interaction	255 (184)
Membrane Transport	28 (24)
**Cellular Processes**	**905 (611)**
Cell Motility	134 (83)
Cell Growth and Death	245 (176)
Transport and Catabolism	411 (280)
Cell Communication	281 (174)
**ORGANISMAL SYSTEMS**	**1,084 (771)**
Immune System	441 (308)
Endocrine System	277 (193)
Circulatory System	112 (73)
Digestive System	187 (126)
Excretory System	98 (64)
Nervous System	265 (186)
Sensory System	34 (24)
Development	160 (109)
Environmental Adaptation	37 (25)
**Total**	**3,699 (2,690)**

* Unique sequences indicate non-redundant sequences involving particular KEGG category.

Well-categorized and annotated transcriptome could serve as important and valuable resources for gene identification and functional analysis of specific traits in common carp genetics and genomics. For instance, 441 transcript contigs associated with immune systems in KEGG analysis had been collected (Table S2). A microarray will be developed with all potential immune-related genes for common carp immunology and disease control research in the collaborator's laboratory.

**Figure 2:**
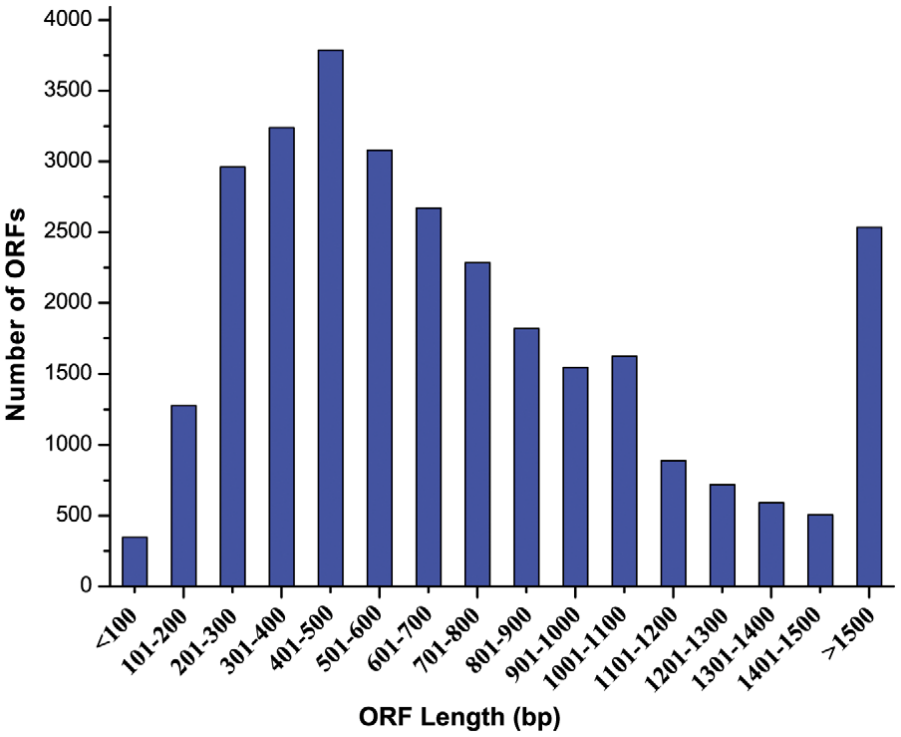
Length distribution of identified ORF from common carp transcriptome assembly.

Of the 36,811 assembled contigs of common carp transcriptome, Open Reading Frames (ORFs) were detected from 29,869 (81.1%) contigs, with an average ORF length of 763 bp ranged from 50 bp to 14,970 bp (Figure 2). The remaining 6,942 contigs contained no ORFs, indicating they are non-coding sequences and likely coming from untranslated regions (UTR). The assembled transcriptome contigs currently served as reference for cSNPs identification from RNA-seq data for multiple common carp strains. ORF analysis would allow to discriminate synonymous and non-synonymous SNPs, and to identify non-sense mutations in common carp.

### Assessment of transcriptome assembly

**Figure 3:**
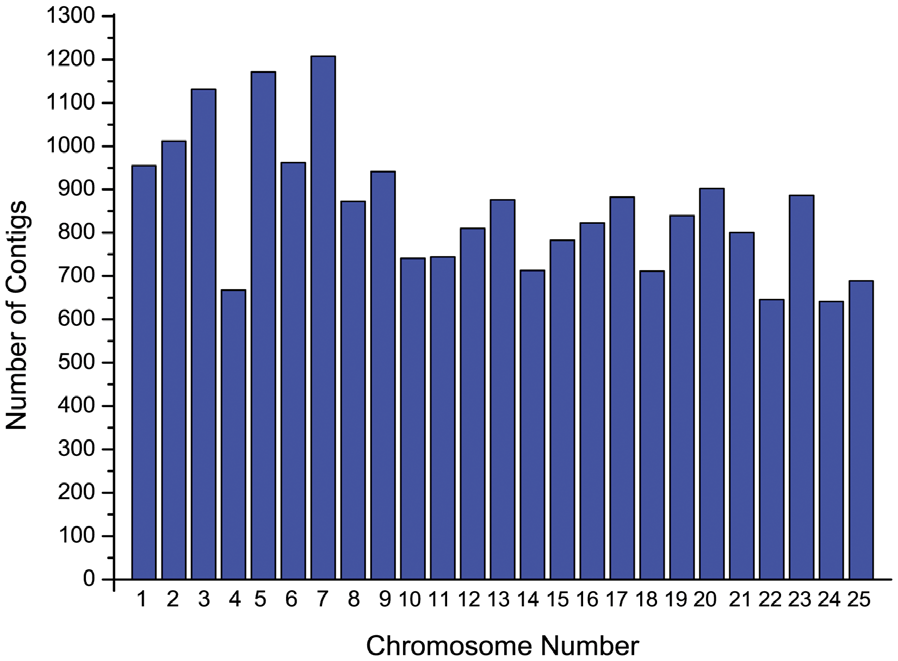
Distribution of common carp transcriptome contig on zebrafish chromosomes.

The assembled contigs of common carp transcriptome were compared with Refseq proteins of zebrafish, fugu (*Takifugu rubripes*), medaka *(Oryzias latipes*) and three-spined stickleback (*Gasterosteus aculeatus*) by using BLASTx program with e-value cutoff of 1E-10. There were 27,693 contigs (75.2%) with 14,554 unique protein hits, 24,371 contigs (66.2%) with 12,536 unique protein hits, 24,501 contigs (66.5%) with 12,471 unique protein hits and 25,025 contigs (70.0%) with 13,147 unique protein hits identified with significant hits on Refseq proteins of zebrafish, teraodon, medaka and three-spined stickleback. The contigs of common carp transcriptome had hits to 47.7% to 54.2% of the unique proteins of zebrafish, tetraodon, medaka and three-spined stickleback (Table 3). Obviously, the closest related zebrafish showed highest similarity to common carp at gene expression level. However, the transcriptome similarity was still relative lower than our expectation. Both zebrafish and common carp belong to Cyprinidae but in different subfamilies distantly according to phylogenetic research [35]. They may only share limited level of similarity. In addition, current research may not be possible to cover the whole transcriptome as only 12 tissues of adult common carp were used. Some rare transcripts may be missed or only collected as singletons during the assembly. For better understanding and characterization of common carp transcriptome, we definitely need a complete set of transcriptome from virtually every tissue across every life stage and every circumstance. To assess transcript distribution in the genome, all contigs of common carp transcriptome were mapped to the complete genome of closest related species, zebrafish (zv9) and all contigs with significant hits were plotted by zebrafish chromosome number as showed in Figure 3.

### Full-length cDNA prediction

**Figure 4:**
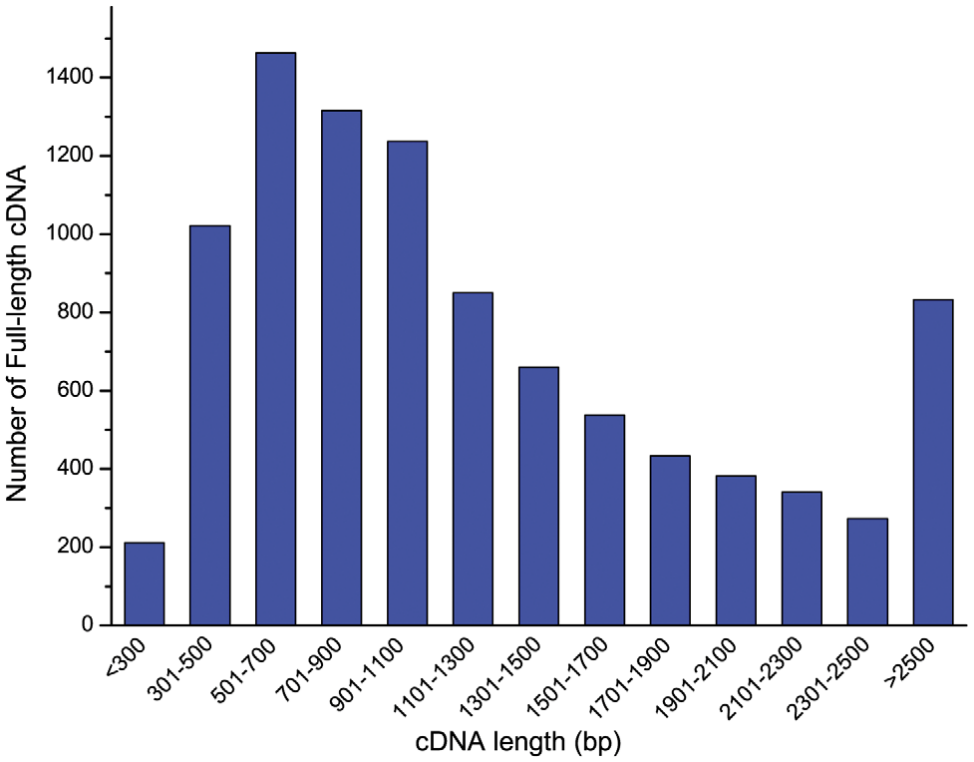
Length distribution of putative full-length cDNAs of common carp.

Full-length cDNAs are important resources for many genetic and genomic researches, including gene duplication analysis, alternative splicing and whole geome sequencing and assembly, etc. To identify potential full-length cDNAs with complete ORF in assembled transcriptom of common carp, all contigs were analyzed by online tool of TargetIdentifier. A total of 9,625 full-length and ORF completely sequenced sequences were identified from the assembly with a cutoff E-value of 1E-5 with sequence length from 201 bp to 9,956 bp (Figure 4). Most of the identified full-length cDNA sequences were shorter than 1.5 kb, suggesting those long full-length cDNA sequences were not easy to be assembled solely with current set of transcriptome data. We may need to combine them with traditional full-length cDNA library and Sanger's sequencing method to collect more full-length cDNA sequences and build a database.

### Repetitive element analysis and microsatellite identification

A total of 2,064 microsatellites were initially identified from 1,730 contigs, including di-, tri-, tetra-, penta- and hexa-nucleotide repeats. After removing the microsatellites without enough flanking sequence for primer design, 1,639 unique sequences with microsatellites had sufficient flanking sequences (50 bp) on both sides of the microsatellites to design primers for genotyping (Table 5).

**Table 5:** Statistics of microsatellites identified from common carp transcriptome.

Total number of contigs	36,811
Microsatellites identified	2,064
Di-nucleotide repeats	9,64
Tri-nucleotide repeats	9,51
Tetra-nucleotide repeats	1,28
Penta-nucleotide repeats	29
Hexa-nucleotide repeats	10
Number of contigs containing microsatellites	1,730
Number of microsatellites with sufficient flanking sequencing for PCR primer design	1,639

The proportion of the repetitive elements in the common carp genome was assessed by using Repeatmasker with Vertebrates Repeat Database. Repeatmasking of the 32,709,720 bp of the carp contig sequences resulted in the detection of 451,894 bp (1.38%) base pairs of repeated sequences. The classification and respective proportion of the identified repetitive elements are shown in Table S3. The most abundant type of repetitive elements in the sequences was DNA transposons (0.42%), mostly hobo-Activator (0.2%), followed by retroelements (0.23%) including LINEs (0.12%), LTR elements (0.1%), and SINEs (0. 01%). Various satellite sequences, low complexity and simple sequence repeats accounted for 0.1%, 0.34% and 0.27% of the base pairs, respectively.

### Conclusion

In this study, the transcriptome of common carp was sequenced with the 454 GS FLX platform with high coverage, and *de novo* assembly was performed with multiple programs and steps. The assembled contigs were then evaluated and functionally annotated by comparing with exiting protein databases of closed related species. The ORF analysis was conducted and a large number of full length cDNA sequences had been identified. In addition, repetitive element analysis was conducted, and cDNA SSRs were identified for future marker development and linkage analysis. Overall, this study on common carp transcriptome developed valuable resource for future genetic or genomic studies on the economically important species.

## Methods

### Ethics Statement

This study was approved by the Animal Care and Use committee of the Centre for Applied Aquatic Genomics at Chinese Academy of Fishery Sciences.

### Biological samples

Gynogenic common carp was generated by using heat shocking treatment of fertilized eggs. Twelve tissues including brain, muscle, liver, intestine, blood, head kidney, trunk kidney, skin, gill, spleen, gonad and heart were dissected and collected from a six-month-old gynogenic common carp. Tissue samples were stored in RNAlater (Qiagen, Hilden, Germeny) at −20°C prior RNA extraction.

### RNA Extraction

Total RNA was extracted from 12 tissues using TRIZOL Kit (Invitrogen, Carlsbad, CA, USA) with manufacturer's instructions. RNA samples were then digested by DNase I to remove potential genomic DNA. Integrity and size distribution were checked with Bioanalyzer 2100 (Agilent technologies, Santa Clara, CA, USA). Equal amounts of the high quality RNA samples from each tissue were then pooled for cDNA synthesis and sequencing.

### cDNA library construction and sequencing

RiboMinus™ Eukaryote Kit for RNA-Seq (Invitrogen) was used to deplete ribosomal RNA from pooled total RNA. Approximately 10 µg of processed total RNA were used for cDNA synthesis using M-MLV RTase cDNA Synthesis kit (TaKaRa, Dalian, China). A total of 10 µg cDNA were used for sequencing library construction at Beijing Institute of Genomics, Chinese Academy of Sciences as manufactory's procedures. Sequencing was then performed using GS FLX Titanium series reagents on Roche Genome Sequencer FLX instrument.

### Sequence data processing and *de novo* assembly

**Figure 5:**
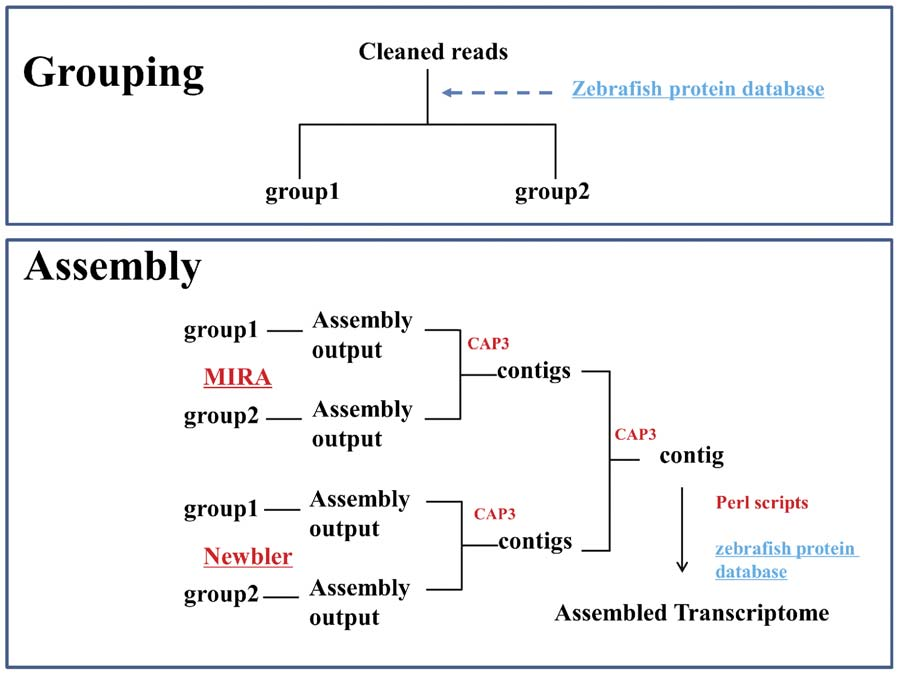
Transcriptome assembly and analysis pipeline.

The raw sequences generated by Roche Genome Sequencer FLX were processed with CLC Genomics Workbench (CLC Bio) and SeqClean (http://compbio.dfci.harvard.edu/tgi/software/). Adaptor sequences were trimmed and low quality reads were removed. To reduce potential complexity during *de novo* assembly, zebrafish protein database were used as reference. Briefly, all cleaned reads were blasted against zebrafish protein database using BLASTx. The reads with high quality hits (reads coverage >80%, identity >60%, and E-value< = 1e-5) were collected as group 1, all other reads were collected as group 2. The reads of two groups were assembled separately with Newbler 2.5 (http://www.454.com) with minimal identity of 95% and minimal length of 100 bp, and MIRA 3.2.1 [36] with default parameters, and then assembled with CAP3 [37] as described previously [38] with modification (Figure 5). Briefly, assembled contigs from both groups with Newbler 2.5 were pooled and re-assembled by CAP3, and assembled contigs from both groups with MIRA were also pooled and re-assembled by CAP3. Then contigs from both batches were merged and re-assembled using CAP3. All collected contigs were BLASTed against zebrafish protein database for identifying coding strand and orientation. For those contigs mapped on same protein reference gene with gaps, the gaps were filled with 20 “X” no matter the real length of the gap.

### Functional annotation

Local BLASTx was performed to align assembled transcriptome contigs to NCBI non-redundant (nr) protein database for functional annotation. The e-value cutoff was set at 1E-10. Gene name was assigned to each contig based on the top BLASTx hit with the highest score. Gene ontology (GO) analysis was conducted on assembled transcriptome sequences by using InterProScan (http://www.ebi.ac.uk/Tools/pfa/iprscan/) and integrated protein databases with default parameters. The GO terms associated with each assembled sequence of common carp transcriptome were then obtained for describing biological processes, molecular functions and cellular components. InterProScan output file was input into BGI WEGO program and GO annotations were plotted (http://wego.genomics.org.cn). All assembled contigs were analyzed by ESTScan to search for ORFs, which could be used to distinguish coding and non-coding sequences.

KEGG pathways were assigned to assembled contigs using the online KEGG Automatic Annotation Server (KAAS) (http://www.genome.jp/tools/kaas/) [39]. The Bi-directional Best Hit (BBH) method was used to obtain KEGG Orthology (KO) assignment.

### Assembly assessment

To compare the similarity to other teleost species, all assembled contigs were compared to Refseq and Ensemble proteins of zebrafish, fugu (*Takifugu rubripes*), medaka *(Oryzias latipes*) and three-spined stickleback (*Gasterosteus aculeatus*). To assess transcript distribution in the genome, all assembled contigs were mapped to zebrafish genome (zv9) by using program BLAT with default parameters.

### Full-length cDNA identification

Putative full-length cDNAs were identified by using online tool TargetIdentifier [25], [40] and comparing to non-redundant protein databases with a cutoff e-value of 10-5. The cDNA sequence was recognized as a full-length cDNA only if the start codon (ATG) and poly (A) tail were identified.

### Repetitive element analysis and microsatellite identification

To identify all repetitive elements in assembled transcriptome of common carp, RepeatMasker was used with Repbase for all vertebrate and zebrafish. A perl-based script Msatfinder V 2.0.9 [41] was used for microsatellite identification from assembled cDNA contigs. The mononucleotide repeats were ignored by modifying the configure file. The repeat thresholds for di-, tri-, tetra-, penta-, hexa-nucleotide motifs were set as 8, 5, 5, 5 and 5 respectively. Only microsatellite sequences with flanking sequence longer than 50 bp on both sides were collected for future marker development.

## Supporting Information

Figure S1 **Length distribution of sequencing reads of common carp transcriptome.**
(TIF)

Figure S2 **Distribution of assembled contig length.**
(TIF)

Table S1 **Assembly statistics for each step.**
(DOC)

Table S2 **Immune-related genes identified from common carp transcriptome.**
(XLS)

Table S3 **Transposable elements in common carp transcriptome.**
(DOC)
