# Supplementary figures and images for: Characterization of Common Carp Transcriptome: Sequencing, De Novo Assembly, Annotation and Comparative Genomics
Source: PLoS One. 2012 Apr 13;7(4):e35152. doi: 10.1371/journal.pone.0035152 (PMC3325976; doi:10.1371/journal.pone.0035152)

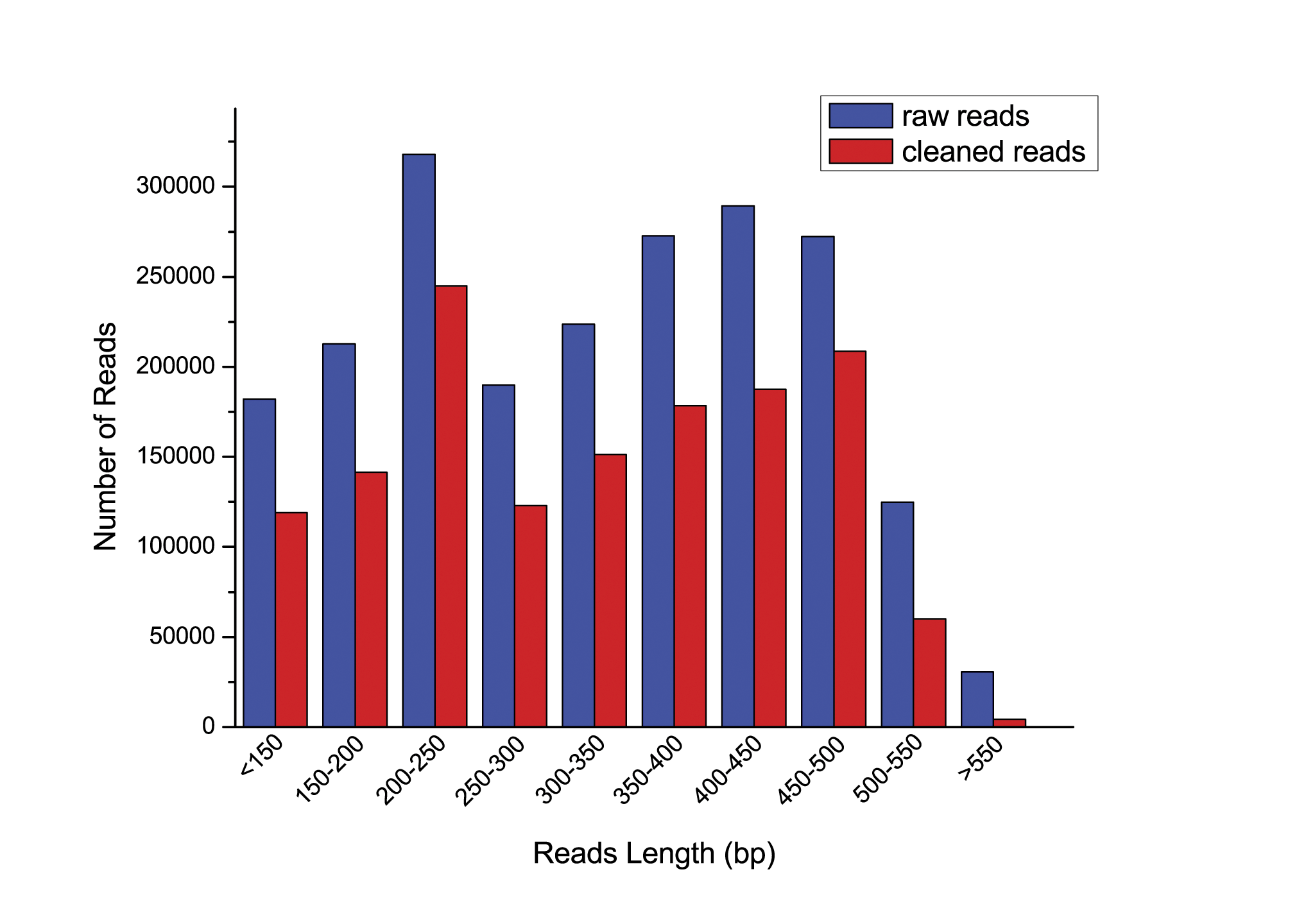

Supplement: Figure S1 — Length distribution of sequencing reads of common carp transcriptome. (TIF) [file pone.0035152.s001.tif]

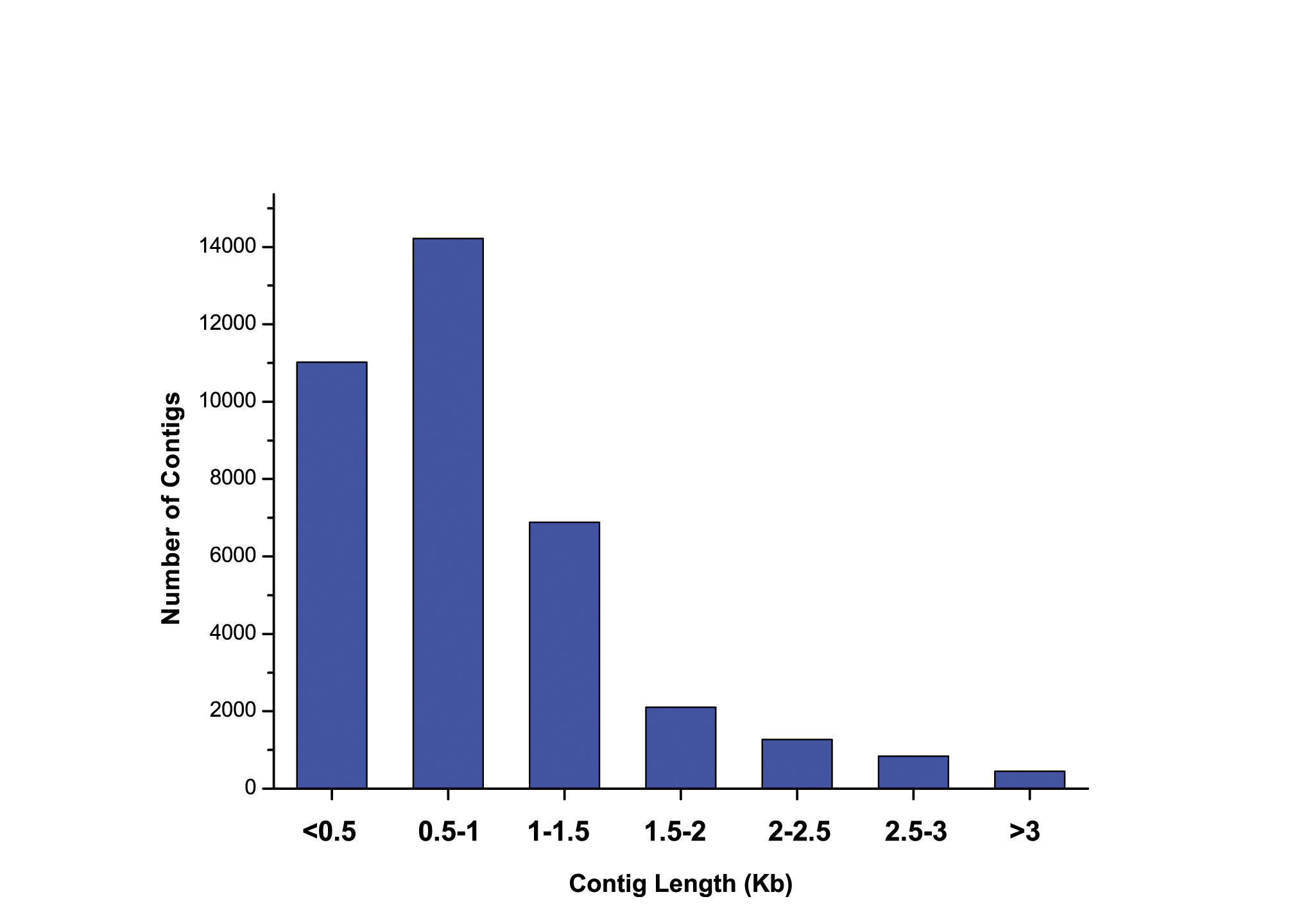

Supplement: Figure S2 — Distribution of assembled contig length. (TIF) [file pone.0035152.s002.tif]
