# Supplementary material for: Characterization of Common Carp Transcriptome: Sequencing, De Novo Assembly, Annotation and Comparative Genomics
Source: PLoS One. 2012 Apr 13;7(4):e35152. doi: 10.1371/journal.pone.0035152 (PMC3325976; doi:10.1371/journal.pone.0035152)
Supplement: Table S1 — Assembly statistics for each step. (DOC) [file pone.0035152.s003.doc]

| Number of clean reads | 1,418,591 | |
| --- | --- | --- |
|  | Group1 | Group2 |
| Number of reads | 778,472 | 640,119 |
|  | | |
| Contigs assembled with MIRA | 40,570 | 74,050 |
| Singleton generated with MIRA | 173,837 | 155,822 |
| Re-assembly with CAP3 | 52,346 contigs, N50 = 714 bp | |
|  | | |
| Contigs assembled with Newbler | 38,278 | 75,644 |
| Singleton generated with Newbler | 3,4005 | 74,475 |
| Re-assembly with CAP3 | 93,631 contigs, N50 = 596 bp | |
|  | | |
| 2^nd^ Round of CAP3 assembly | 41,509 contigs, N50 = 809 bp | |

Supplementary Table 1. Assembly statistics for each step
