# Supplementary material for: Characterization of Common Carp Transcriptome: Sequencing, De Novo Assembly, Annotation and Comparative Genomics
Source: PLoS One. 2012 Apr 13;7(4):e35152. doi: 10.1371/journal.pone.0035152 (PMC3325976; doi:10.1371/journal.pone.0035152)
Supplement: Table S3 — Transposable elements in common carp transcriptome. (DOC) [file pone.0035152.s005.doc]

|  |  | number of elements | length occupied  (bp) | Percentage of  Sequence (%) |
| --- | --- | --- | --- | --- |
| Retroelements |  | 490 | 75,715 | 0.23 |
|  | SINEs | 57 | 4,767 | 0.01 |
|  | Penelope | 2 | 112 | 0.00 |
|  | LINEs | 173 | 37,664 | 0.12 |
|  | L2/CR1/Rex | 131 | 32,238 | 0.1 |
|  | R1/LOA/Jockey | 9 | 1,137 | 0 |
|  | R2/R4/NeSL | 1 | 83 | 0 |
|  | RTE/Bov-B | 6 | 1,237 | 0 |
|  | L1/CIN4 | 23 | 2,811 | 0.01 |
|  | LTR elements: | 260 | 33,284 | 0.1 |
|  | BEL/Pao | 11 | 2,423 | 0.01 |
|  | Ty1/Copia | 0 | 0 | 0 |
|  | Gypsy/DIRS1 | 85 | 18,458 | 0.06 |
|  | Retroviral | 128 | 8,904 | 0.03 |
|  |  |  |  |  |
| DNA transposons |  | 1290 | 137,121 | 0.42 |
|  | hobo-Activator | 668 | 65,046 | 0.2 |
|  | Tc1-IS630-Pogo | 106 | 16,352 | 0.05 |
|  | En-Spm | 103 | 6,494 | 0.02 |
|  | PiggyBac | 14 | 2,026 | 0.01 |
|  | Tourist/Harbinger | 44 | 6,304 | 0.02 |
|  | Other(Mirage,  P-element, Transib) | 1 | 144 | 0 |
| Unclassified |  | 22 | 1,407 | 0 |
| Small RNA |  | 55 | 7,547 | 0.02 |
| Satellites |  | 238 | 32,720 | 0.1 |
| Simple repeats |  | 2066 | 89,072 | 0.27 |
| Low complexity |  | 2425 | 110,948 | 0.34 |
